# Axial wall angulation for rotational resistance in a theoretical‐maxillary premolar model

**DOI:** 10.1002/cre2.229

**Published:** 2019-08-30

**Authors:** John F. Bowley, Po Lee, Wen‐Fu Thomas Lai

**Affiliations:** ^1^ Dental Service (523/160) VA Boston Healthcare System Jamaica Plain Massachusetts; ^2^ Restorative Dentistry and Biomaterials Sciences Harvard School of Dental Medicine Boston Massachusetts; ^3^ Resident, Periodontology Residency Program Tufts University School of Dental Medicine Boston Massachusetts; ^4^ Center for Nano‐tissue Engineering & Imaging Research Taipei Medical University Hospital Taipei Taiwan, ROC; ^5^ McLean Imaging Center McLean Hospital/Harvard Medical School Belmont Massachusetts

**Keywords:** fixed restoration stability, premolar‐sized tooth model, preparation surface area, rotational resistance form, supplemental groove

## Abstract

**Objectives:**

The aim of this study was to determine the influence of short base lengths and supplemental grooves on surface area and rotational resistance in a simulated‐maxillary premolar.

**Materials and Methods:**

Trigonometric calculations were done to determine the total surface area with and without supplemental grooves. Additional computations were done to determine the maximum wall angle needed to resist rotation displacement in a premolar‐sized model. Wall heights of 3.0, 4.0, and 5.0 mm were used in the surface area and rotational axis computations. The rotational axis was located on the lingual restoration margin to produce a buccal‐to‐lingual rotational displacement.

**Results:**

Total surface area decreased with increasing four‐wall taper levels from 2° to 18° and decreasing preparation heights from 5 to 3 mm. Significant surface area improvements were found with the supplemental use of mesial and distal axial grooves compared with the same condition without grooves in all taper levels and preparation height categories. Resistance to rotational displacement was determined to occur at only at very low levels of opposing wall taper angles. The use of supplemental grooves on mesial and distal axial walls significantly improved both total surface area and rotational resistance.

**Conclusions:**

The vertical wall taper angles, preparation heights, and supplemental grooves play a role in resistance form and restoration stability.

## 1 INTRODUCTION

The literature has demonstrated many factors related to the stability of a fixed restoration in function to maintain its position in resistance to rotational forces. The components of resistance to rotation include vertical wall angulation of the prepared tooth, wall height, total surface area, preparation adjunctive features, vertical height location of the rotational axis, and the base length from the rotational axis (Tiu, Al‐Amleh, Waddell, & Duncan, 2015). Historically, for many years, students have been taught preparation standards as found textbooks (Rosenstiel, Land, & Fujimoto, 2016).

A classic literature review of this topic (Goodacre, Campagni, & Aquilino, 2001) has recommended a narrow range of ideal axial wall taper angles from the long axis of the tooth preparation. However, the maximal wall angulation needed to resist rotation of the restoration around an axis has been shown to be influenced by other factors (Bowley, Ichim, Kieser, & Swain, 2013; Bowley, Kaye, & Garcia, 2017; Bowley & Kieser, 2007; Bowley & Lai, 2007).

One of the most basic considerations that influences the maximal axial wall taper to provide rotational resistance is the vertical height of the tooth preparation. Taller tooth preparation height provides greater resistance to rotation at larger angles of taper compared with shorter tooth preparation height for the same tooth size (Bowley & Kieser, 2007). Surface area of the tooth preparation has also been shown to be indirectly associated with resistance to rotation (Bowley & Lai, 2007). As the axial wall taper angle increases, the total surface area decreases, presumably, reducing the area for luting agent‐restoration interaction. Supplemental grooves have been recommended to improve poor or marginal resistance form (Goodacre et al., 2001) with one study demonstrating these adjuncts increase the surface area of the preparation (Bowley & Lai, 2007).

An additional modifier associated with tooth size is the influence of base width of the base; a recent investigation (Bowley et al., 2017) has shown that a larger distance from the resisting wall to the rotational axis requires significantly lower wall taper angulations for resistance compared with the single molar‐sized tooth form. Lastly, the relative height of the preparation rotational axis has been shown to significantly influence preparation resistance to rotation. Two studies (Bowley et al., 2017; Bowley, Sun, & Barouch, 2004) have shown that shortening the height of the rotational axis relative to the height of the resisting wall requires much lesser limited taper angles compared with the same preparation in which the opposing finish line and the rotational axis are at the same, even height levels.

As cited above, vertical preparation height and base widths have been investigated in previous studies (Bowley et al., 2017; Bowley & Kieser, 2007) in the molar‐ and Fixed Partial Denture (FPD)‐sized restorations. However, these factors have not been assessed in the smaller, rectangular‐tooth form of maxillary premolars. The purpose of the present study is to determine the contribution of smaller tooth base size of the premolar model as well as vertical height to axial wall angulations needed to provide resistance to rotation.

## 2 MATERIALS AND METHODS

### 2.1 Simulated‐premolar tooth form, geometric model

**Figure 1:**
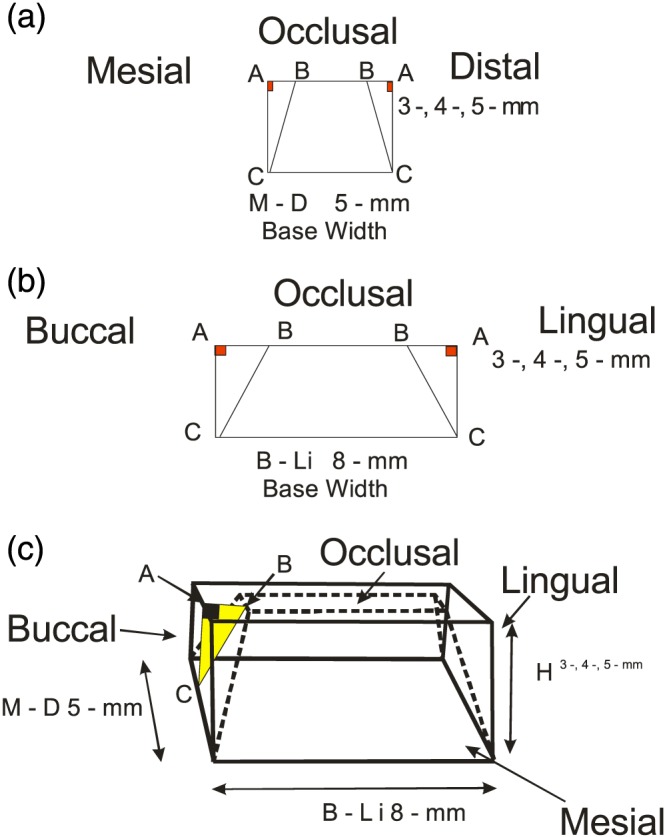
Illustrates the simulated maxillary premolar as rectangular cube: (a) mesial–distal base width 5 mm (M–D B^5 mm^), (b) bucco–lingual base width 8 mm (B–Li B^8 mm^), and (c) three vertical height categories 3, 4, and 5 mm (H^3, 4, 5 mm^)

A geometric figure served as the simulated‐tooth form in a theoretical experimental model system; the experimental‐tooth form with axial wall preparations was a truncated pyramid, and the base lengths and widths approximated the size of a maxillary premolar. In the preoperative condition, the tooth form prior to axial wall preparation was represented by a rectangular cube. This experimental cubic tooth form was manipulated to simulate a crown preparation with four‐angled vertical walls and a flat occlusal surface; the experimental‐premolar tooth can be seen in Figure 1.

### 2.2 Height categories and base widths

The horizontal base‐width and base‐length dimensions were 5 mm in mesial–distal width (M–D B^5 mm^) and 8 mm in bucco–lingual length (B–Li B^8 mm^). The model had three vertical height categories—3.0, 4.0, and 5.0 mm (H^3, 4, 5 mm^)—with trial manipulations within the experimental model system. These three height categories, H^3, 4, 5 mm^, served as an independent variable in this investigation. According to the literature (Goodacre et al., 2001), all three‐tooth height categories, H^3, 4, 5 mm^, used in this investigation would be considered acceptable height levels for this premolar‐sized tooth with these dimensions.

### 2.3 Axial wall taper categories

The rectangular cube had four levels of axial wall inclinations to simulate a tooth preparation with a narrowing of the occlusal surface as the axial wall angulations increased. The simulated preparation had four categories of axial wall inclination, 2°, 6°, 12°, 18°, in the axial walls mesial (M), distal (D), buccal (B), and lingual (Li). These levels of axial wall inclination (M, D, B, and Li^2°, 6°, 12°, 18°^) served as an independent variable throughout the investigation.

### 2.4 *α* ^1^ Total surface area

Each of the angulation categories with four‐axial wall inclinations transformed the rectangular cube into a truncated pyramid with known dimensions. A series of trigonometric analyses were conducted to determine the total surface area of the simulated preparations at each taper angulation, 2°, 6°, 12°, 18°, within each height category, H^3, 4, 5 mm^. The calculation of the total surface area in square millimeters, four‐axial walls and an occlusal surface, served as a dependent variable *α* ^1^, formula derivations in molar‐sized tooth model published in Bowley and Lai (2007). The total surface area data, *α* ^1^, at each of the four‐axial wall angulation categories in three height categories H^3, 4, 5 mm^ can be seen in Table 1.

**Table 1:** Total surface area, as *α* ^1^, four vertical walls and occlusal surface of simulated prepared premolar tooth form B^5 mm^ M–D × B^8 mm^ B–Li widths: Total surface area with increasing axial wall taper angulations compared with unaltered rectangular cube as *α* ^1^ in mm^2^ at 2°, 6°, 12°, and 18° for categories H^3, 4, 5 mm^

Four‐wall total surface area axial taper (°)		Four walls + occlusal *α* ^1^ (mm^2^)
H^3 mm^		
0°	Rectangular block	118.0
2°		116.7
6°		114.4
12°		111.7
18°		109.7
H^4 mm^		
0°	Rectangular block	144.0
2°		142.3
6°		139.3
12°		135.8
18°		133.5
H^5 mm^		
0°	Rectangular block	170.0
2		167.8
6°		164.1
12°		160.0
18°		157.4

### 2.5 *α* ^2^ Surface area gain supplemental M‐ and D‐grooves

Additional surface area calculations, represented as *α* ^2^ values in square millimeters, were the surface area of two supplemental grooves in the M‐ and D‐axial walls as a second dependent variable. The two supplemental grooves were experimentally placed M‐ and D‐axial walls in all four 2°, 6°, 12°, 18° taper categories in all three H^3, 4, 5 mm^ height categories. The total surface area of both supplemental grooves was determined by trigonometric methods. The final surface area gained was the total surface area of both grooves minus the occlusal and axial wall surface area lost in groove placement; these net gain values were *α* ^2^ data and can be seen in Table 2; *α* ^2^ formula derivations in the molar‐sized tooth model have been published in Bowley and Lai (2007).

**Table 2:** Total area of two grooves with axial wall and occlusal surface loss in placement of two grooves in premolar 5 mm M–D × 8 mm B–Li widths, four‐axial wall taper categories 2°, 6°, 12°, and 18° and three height categories H^3, 4, 5 mm^ *α* ^2^ as mm^2^ net area gain

Three height categories (mm) and four‐axial wall taper categories (°)	Surface area lost groove placement (mm^2^)	Two‐groove surface area (mm^2^)	*α* ^2^ Net gain (mm^2^)
H^3 mm^			
2°	−11.365	28.9	+17.6
6°	−11.360	32.1	+20.8
12°	−11.344	35.1	+23.8
18°	−11.319	38.7	+27.4
H^4 mm^			
2°	−15.684	38.0	+22.3
6°	−15.676	41.6	+25.9
12°	−15.649	46.0	+30.4
18°	−15.603	47.7	+32.0
H^5 mm^			
2°	−19.244	47.9	+28.6
6°	−19.231	52.7	+33.5
12°	−19.188	59.1	+39.9
18°	−19.117	66.3	+47.2

### 2.6 *α* ^3^ Maximal buccal axial wall rotational resistance

The *α* ^3^ values, as the third dependent variable shown in Table 3, represented the trigonometric calculation of the maximum axial wall angulation needed to provide rotational resistance around the lingual axis. Three *α* ^3^ levels were calculated, one for each H‐category, H^3, 4, 5 mm^. The *α* ^3^ values were calculated according to the formula:

$$\alpha^{3}=½\;\left[\text{ASIN}\;\left(H^{3,4,5\;\mathrm{mm}}÷B^{8\;\mathrm{mm}}\right)\right]$$

**Table 3:** Premolar 5 mm M–D × 8 mm B–Li widths with maximal rotational resistance wall opposite rotational axis for three height categories H^3, 4, 5 mm^ (*α* ^3^) and two M‐ and D‐grooves improvement (*α* ^4^)

	Maximal opposing wall rotational resistance angle (*α* ^3^ degrees)	Base length (mm)	Groove placement^a^ three 172‐tapered bur (*α* ^4^ degrees)	Base^a^ length (mm)
H^3 mm^	≤11.0°	8	3°	3.41
H^4 mm^	≤15.0°	8	3°	3.41
H^5 mm^	≤19.3°	8	3°	3.41

a Three degrees represents one side of 172‐tapered bur with 6° taper overall and tooth base size midpoint of 8‐mm base = 4 mm with center point of bur tip 4–0.59 mm as radius of small tip circle so 3° wall‐opposing rotational axis location at 3.41 mm.

These three rotational resistance values in were done for the truncated pyramid in each H‐category without axial groove supplements; *α* ^3^ formula derivations have been published in Parker, Gunderson, Gardner, and Calverley (1988).

### 2.7 *α* ^4^ Rotational resistance M‐ and D‐grooves

The fourth dependent variable, *α* ^4^, was the level of rotational resistance provided by the supplemental axial grooves; *α* ^4^ formula derivations have been published in Parker et al. (1988). The *α* ^4^ values for each H‐category were calculated with the same formula as *α* ^3^ above but a shorter base length B–Li B^3.41 mm^:

$$\alpha^{4}=½\;\left[\text{ASIN}\;\left(H^{3,4,5\;\mathrm{mm}}÷B^{3.41\;\mathrm{mm}}\right)\right]$$

The groove placement was located at the 4‐mm B–Li B^8 mm^ midpoint with the 172‐tapered bur; this position would locate the center of the bur over this midpoint with the 3° Li‐rotational resisting wall at 4 mm minus the radius of small bur tip, 0.59 mm. This would place the 3° lingual wall of the groove at 3.41 mm from the lingual rotational axis. Thus, the Li‐wall of both M‐ and D‐grooves served as the resisting wall with a reduction of the length from axis of rotation from 8 to 3.41 mm and reducing the angulation of the resisting wall from H^3 mm^ 11.0°, H^4 mm^ 15.0°, and H^5 mm^ 19.3° to 3°.

### 2.8 Stepwise computations with trigonometric formula

**Figure 2:**
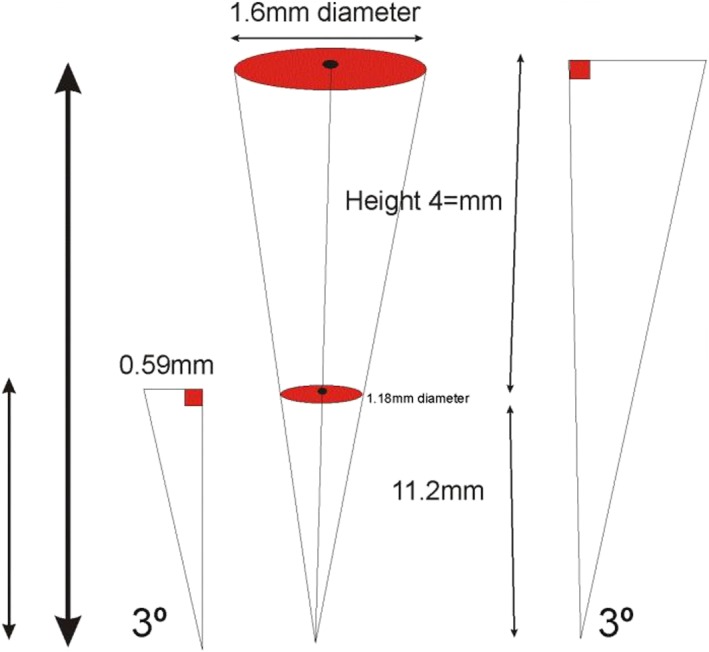
Large and small cones related to calculation of the 172‐tapered bur's diameter of the preparation at the occlusal surface level; known values are bur taper 6° convergence angle for 3° groove wall angulation and bur tip diameter 1.16 mm

**Figure 3:**
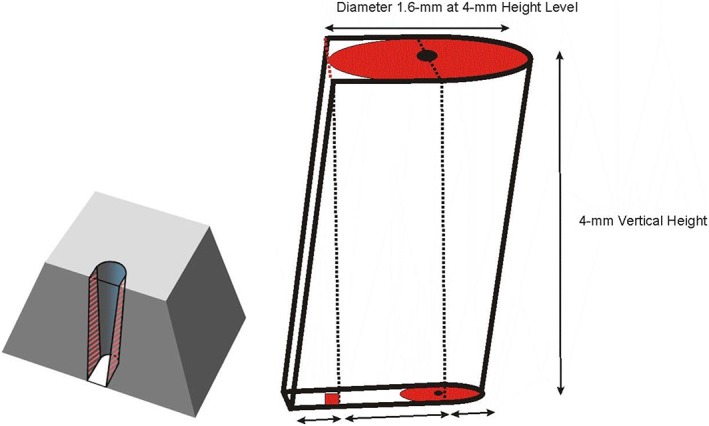
Calculation of one‐half conical surface area, lateral wall rectangles, and right triangles

**Figure 4:**
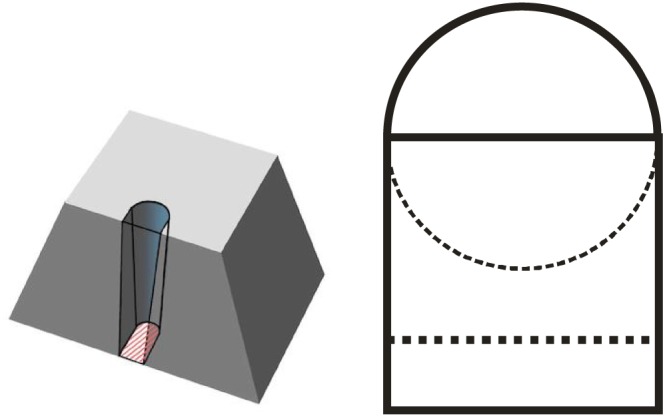
Calculation of the area of groove floor

**Figure 5:**
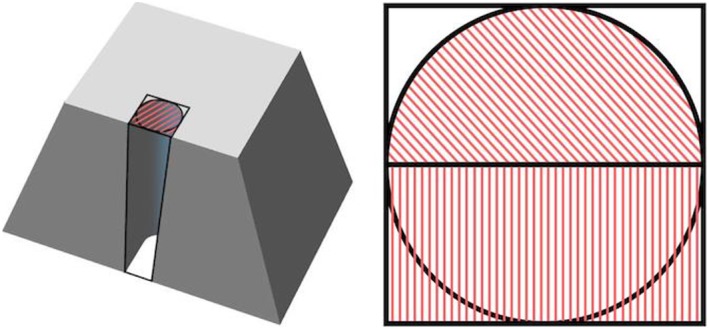
Calculation of external surface area lost from groove cut on occlusal surface

**Figure 6:**
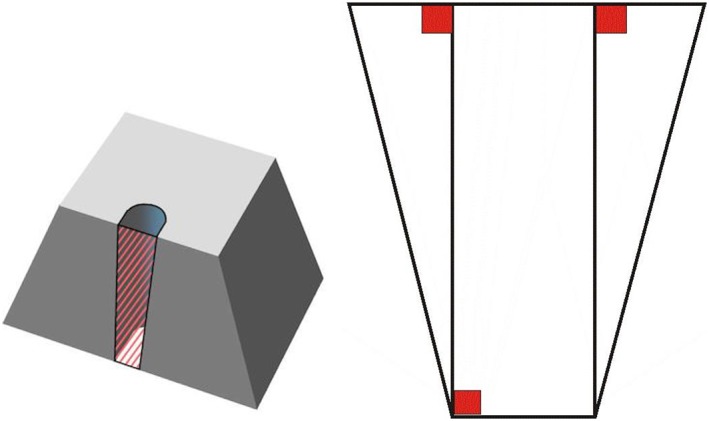
Calculation of external surface area lost from groove cut on axial wall = 2 right triangles + 1 rectangle

**Figure 7:**
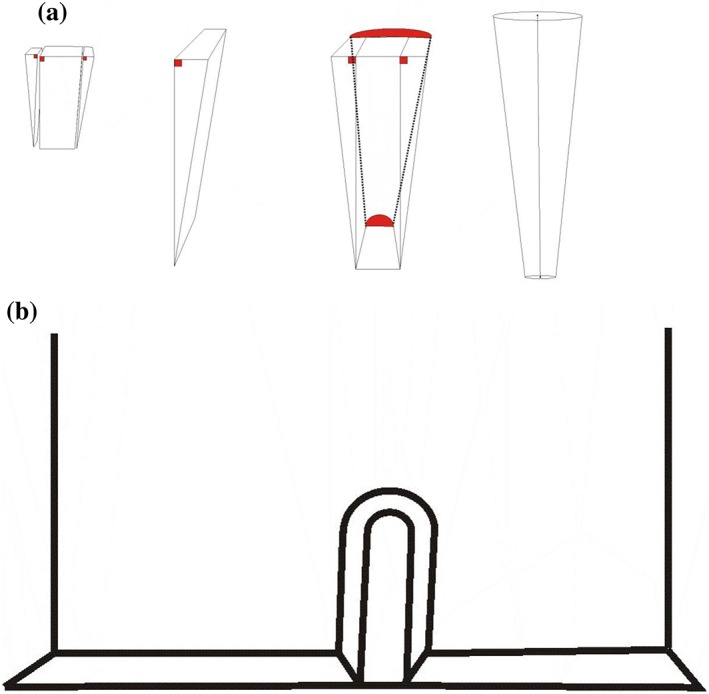
Miscellaneous illustrations of groove components and visual overview looking from occlusal view. (a) Illustrates various components of the supplemental groove area computations. (b) Illustrates the supplemental groove from occlusal view, looking down on the occlusal surface and axial wall

The total surface area and two‐groove area calculations can be seen in the stepwise formulas with illustrations in Appendix A and Figures 1, 2, 3, 4, 5, 6, 7. These formulae derivations were based on the Pythagorean Theorem and trigonometric functions within right triangles with two‐known values to be used in the calculation of a third‐unknown value (Lial, Schneider, & Hornsby, 2004).

## 3 RESULTS

### 3.1 *α* ^1^ Total surface area

The simulated‐maxillary premolar model demonstrated a decreasing total surface area *α* ^1^ values as preparation M‐, D‐, B‐, and Li‐wall tapers increase from 2° to 18° and as vertical preparation heights decreased from H^5.0 mm^ to H^3.0 mm^, as can be seen in Table 1. Total surface area *α* ^1^ values of the uncut rectangular blocks in each height category were as follows:

$$\begin{array}{l} H^{3.0‐\mathrm{mm}}\left(5\;\mathrm{mm}\times 3\;\mathrm{mm}\right)+\left(5\;\mathrm{mm}\times 3\;\mathrm{mm}\right)+\left(8\;\mathrm{mm}\times 3\;\mathrm{mm}\right)+\left(8\;\mathrm{mm}\times 3\;\mathrm{mm}\right)+\left(8\;\mathrm{mm}\times 5\;\mathrm{mm}\right)=118.0\;{\mathrm{mm}}^{2}\\ H^{4.0‐\mathrm{mm}}\left(5\;\mathrm{mm}\times 4\;\mathrm{mm}\right)+\left(5\;\mathrm{mm}\times 4\;\mathrm{mm}\right)+\left(8\;\mathrm{mm}\times 4\;\mathrm{mm}\right)+\left(8\;\mathrm{mm}\times 4\;\mathrm{mm}\right)+\left(8\;\mathrm{mm}\times 5\;\mathrm{mm}\right)=144.0\;{\mathrm{mm}}^{2}\\ H^{5.0‐\mathrm{mm}}\left(5\;\mathrm{mm}\times 5\;\mathrm{mm}\right)+\left(5\;\mathrm{mm}\times 5\;\mathrm{mm}\right)+\left(8\;\mathrm{mm}\times 5\;\mathrm{mm}\right)+\left(8\;\mathrm{mm}\times 5\;\mathrm{mm}\right)+\left(8\;\mathrm{mm}\times 5\;\mathrm{mm}\right)=170.0\;{\mathrm{mm}}^{2} \end{array}$$

In the H^3 mm^ category, the *α* ^1^ values revealed the lowest values of the investigation with the 18° per wall at 109.7 mm^2^ compared with the greatest value within this H‐category as 2° ideal at 116.7 mm^2^; between these two extremes, the 6° and 12° levels showed 114.4 and 111.7 mm^2^, respectively. In the H^4 mm^ category, the *α* ^1^ values revealed greater overall area values compared with the H^3 mm^ levels with the lowest value in 18° per wall at 133.5 mm^2^ compared with the greatest value in the 2° ideal at 142.3 mm^2^; between these two extremes, the 6° and 12° levels showed 139.3 and 135.8 mm^2^, respectively. The H^5 mm^ category followed a similar trend to other two H‐categories with the highest the *α* ^1^ values found in this investigation. The lowest H^5 mm^ *α* ^1^ value was found in the 18° group at 157.4 mm^2^ and the greatest value in the 2° ideal at 167.8 mm^2^ with the 6° and 12° levels at 164.1 and 160.7 mm^2^, respectively.

### 3.2 *α* ^2^ Surface area gain two supplemental M‐ and D‐grooves

The *α* ^2^ data (see Table 2) showed a net gain of total surface area in all taper angulations and all height groupings with two supplemental grooves; the two supplemental grooves were introduced to the M‐ and D‐axial walls to the dimensions of a 172‐tapered fissure bur. Each groove preparation produced an axial and occlusal surface area loss from the groove surface preparation procedure; these initial surface area losses were H^5 mm^ 2° as the greatest loss at −19.244 mm^2^ and H^3 mm^ 18° as the lowest amount at −11.319 mm^2^. All other taper and height categories were intermediate to these two extremes. This step was a component of the *α* ^2^‐calculation values as a subtraction from the final total.

The total groove surface area for two supplemental grooves was calculated as a net gain in the *α* ^2^ values once the net loss of the preparation surface area component was subtracted. The total surface area net gain for both grooves was calculated with the greatest surface area gained in the H^5 mm^ 18° preparation category with +47.2 mm^2^ net gain. Additional 2°, 6°, and 12° preparation category values in were found at +28.6, +33.5, and +39.9 mm^2^. Lesser *α* ^2^ values were found in the H^3, 4 mm^ categories with similar trends as the highest values at 18° preparation categories at +32.0 and +27.4 mm^2^, respectively. The other taper categories 2°, 6°, and2° in the H^3, 4 mm^ groupings had lesser net gain values in the range of +20.8 to +30.4 mm^2^.

### 3.3 *α* ^3^ Minimal B‐axial wall angulation rotational resistance

The *α* ^3^ values revealed the maximal B‐axial wall angulations allowable to resist the rotation of the restoration around the lingual axis; each height category had a maximal allowable B‐wall angulation that would need to be attained for rotational resistance H^3.0 mm^ 11.0°, H^4.0 mm^ 15.0°, and H^5.0 mm^ 19.3°. Any B‐wall angulation, greater than these values, would allow rotation of the restoration with an absence of physical barrier as resistance other than the luting agent itself.

### 3.4 *α* ^3^ Minimal B‐axial wall angulation rotational resistance

The *α* ^3^ values revealed the maximal B‐axial wall angulations allowable to resist the rotation of the restoration around the lingual axis; each height category had a maximal allowable B‐wall angulation that would need to be attained for rotational resistance H^3.0 mm^ 11.0°, H^4.0 mm^ 15.0°, and H^5.0 mm^ 19.3°. Any B‐wall angulation, greater than these values, would allow rotation of the restoration with an absence of physical barrier as resistance other than the luting agent itself.

### 3.5 *α* ^4^ Two‐groove rotational resistance

The placement of two supplemental grooves with the tapered bur dimensions of a 172‐tapered fissure bur at the midpoint of the M‐ and D‐axial walls created a 3° tapered groove wall; a component of the tapered groove provided a 3° vertical wall for rotational resistance. This wall was located at a calculated point 3.41 mm from the rotational lingual axis in all height categories, H^3, 4, 5 mm^. All three height categories had a 3° Li‐wall within each of the M‐ and D‐axial grooves; each of these 3° Li‐walls were well below the *α()*‐maximal angles of H^3.0 mm^ 11.0°, H^4.0 mm^ 15.0°, and H^5.0 mm^ 19.3°. In addition, the two 3° B‐walls of both grooves provided a second wall of rotational resistance; this second wall of low angulation would not normally be available in preparations with only four vertical axial walls.

## 4 DISCUSSION

This investigation has demonstrated a general trend associated with shorter base widths in the maxillary simulated‐premolar model system. The trend was decreasing preparation surface areas with increasing axial four‐wall angulations and shorter vertical preparation heights. These two factors were found to produce a decrease in total surface area; in addition, these two factors were found to be additive when looked at together. Essentially, very low tooth preparation surface area values with the lowest α()‐dependent variable level in 18° axial wall taper grouping paired with the shortest vertical preparation height, H^3 mm^. Presumably, maximizing total tooth preparation area would allow the maximum amount of tooth‐luting agent interaction with improved restoration stabilization during masticatory loading; this process would be expected to contribute to a longer restoration functional lifetime.

A summary of the various factors to influence restorations stability in function include total surface area, axial wall inclination, vertical preparation height, tooth base width, preparation supplemental adjuncts, luting agents, dentine or core materials, and masticatory forces. The current investigation looked at axial angulation, vertical height, base width, and grooves in the absence of these other factors. The objective of theoretical rotational resistance form studies has been to maximize the preparation attributes to minimize or offset the effects of other negative factors such as masticatory forces of occlusion and luting agent function.

Five experimental laboratory investigations represent much of the in vitro data on resistance form as axial wall angulation and grooves in crown restorations (Cameron, Morris, Keesee, Barsky, & Parker, 2006; Lu & Wilson, 2007; Proussaefs, Campagni, Bernal, Goodacre, & Kim, 2004; Roudsari & Satterthwaite, 2011; Trier, Parker, Cameron, & Brousseau, 1998). Trier et al.'s, 1998 investigation looked at clinically dislodged crowns in patients to determine the proportion of resistance in these failed‐restorations; a statistically significant number had inadequate resistance in premolars and molars.

Proussaefs et al.'s (2004) in vitro study of axial wall angulation and groove supplementation was evaluated in a stress‐to‐failure model with a glass ionomer luting agent. This investigation demonstrated the failure levels of 136 kg in the control group compared with 129–193 kg, the grooves and boxes groups compared with 313 kg for 4° modification/improvement group. The use of boxes and grooves did not improve the results in this investigation, but the force levels in all groups was very high compared with normal masticatory loads.

Cameron et al.'s (2006) in vitro study performed a cyclical loading of crowns to stainless steel dies at lower force levels, 2 kg, with glass ionomer cement to varying axial wall angulations from 4° to 32°. No groove supplementation was utilized in this investigation, but the time of resistance to dislodgement was significantly shorter in the specimen with axial wall taper angles >12°. The Lu and Wilson (2007) in vitro study evaluated 50° total convergence angulation (TOC) or 25° per wall in extracted human molar teeth. All specimens were prepared to the same level of vertical height, axial wall taper, and height to base ratios of 0.3 with and without one or two pairs of grooves. All samples were stressed at an oblique angle to failure in the range of 23 to 331 N; the lowest failure levels occurred in the samples axial walls at 25° without grooves and the highest with two pairs of grooves.

Roudsari and Satterthwaite's (2011) study was the most recent laboratory study and utilized ivorine teeth prepared to 20° TOC; the two experimental categories utilized on group with groove placement and an another with reduced 4° TOC in the gingival aspect at 1.5 mm from the finish line. The samples were metal dies representing tooth preparation with CrCo‐alloy restorations and zinc phosphate luting agent. All samples were stressed to dislodgment failure. The improved 4° axial wall supplement stress failure was 221 N compared with 157 N among the groove preparations group, which was statistically significant. These four studies have conflicting results with only one study (Lu & Wilson, 2007) found that groove supplementation improved resistance form failure.

It is difficult to compare the current investigation to any of these studies due to use of luting agent, differing levels of axial wall angulation, variability of different tooth model modulus of elasticity, and so on. The potential importance of the contribution of the tooth's modulus of elasticity can be seen in two finite element analysis (FEA) studies (Bowley et al., 2013; Wiskott, Krebs, Scherrer, Botsis, & Belser, 1999); both studies revealed a rotational axis located midway between buccal and lingual axial walls below the restoration in the root area. This axis location in both studies was different than the current investigation and would be expected to be more favorable to restoration stability. Although neither investigation stressed the system to failure, the axis location is an important consideration.

In contrast to the above cited in vitro laboratory studies, two FEA studies have demonstrated that the restoration‐tooth combination under angular load caused a bending of the system. The bending of the tooth‐restoration system increased with increasing axial wall angulation levels due to decreasing amounts of tooth structure overall. Although this FEA investigation did not generate restoration failure, the loading level of 200 N was significantly higher than the 2 kg of the in vitro investigation. This consideration, tooth system rigidity and flexure under load, may be a significant factor and was not considered in the current investigation or the in vitro laboratory studies.

The most significant findings of the present investigation were the significant contribution of the supplemental axial grooves. The total preparation area increased with the use of two proximal wall grooves as demonstrated in the α^2^ values. In addition, these supplements provided significantly improved rotational resistance as shown with comparison of the *α* ^4^ versus *α* ^3^ values. The placement of two supplemental groove in M‐ and D‐axial walls categories improved both rotational resistance and total surface area in all categories including the worst case, H^3 mm^ at 18°.

As has been shown in other investigations (Bowley et al., 2017; Bowley & Kieser, 2007), the present study's *α* ^1^ data showed decreases in total preparation surface area with increasing axial wall taper levels; however, the present investigation's differences between axial wall angulation categories at the same vertical height were more moderate compared with previous investigations (Bowley et al., 2017; Bowley & Kieser, 2007). The trigonometric analyses showed gradual reductions of surface area with each increment of taper increase from 2° to 18°, at the same vertical preparation heights. The *α* ^1^ data comparison between height categories, H^3, 4, 5 mm^, revealed substantial differences with changes in vertical height.

As in previous investigations (Bowley et al., 2017; Bowley & Kieser, 2007), this independent variable, preparation height was a significant factor in preparation resistance form. The range of differences based on height at the same taper level was 109.7 mm^2^ in H^3 mm^ versus 133.5 mm^2^ in H^4 mm^ versus 157.4 mm in H^5 mm^ in the 18° samples in the three height categories; this amounts to a 50‐mm^2^ difference or 50% more surface area in the worst‐case scenario of H^3 mm^ versus H^5 mm^ at 18°. This evidence demonstrates the significant improvement with extending the preparation height by 2 mm.

Dependent variable *α* ^1^, as the total surface area of a simulated tooth preparation, has been previously investigated in simulated molar‐sized and FPD‐sized tooth form as truncated pyramids with larger base widths (Bowley et al., 2017; Bowley & Kieser, 2007). Dependent variable *α* ^2^, the net gain of preparation surface area with groove supplementation, is a new factor in the literature and was derived from standard geometric and trigonometric calculations with cones, right triangles, and other components. Dependent variables *α* ^1, 3, 4^ were also determined with geometric and trigonometric methods to determine rotational resistance of the axial wall opposite the axis of rotation. The three dependent variables have appeared in the literature in other studies (Bowley & Kieser, 2007; Parker et al., 1988).

## 5 CONCLUSIONS

The maxillary premolar tooth preparation has the smallest base widths of restorations on posterior teeth, but the preparation design features of vertical height, axial wall angulation, and groove supplementation can improve the rotational resistance form of this tooth form.

## CONFLICT OF INTEREST

We declare that we have no competing financial interests.

## FUNDING INFORMATION

This study was supported, in part, by *Fulbright Specialist Program Grant*, American Institute Taiwan, and Taipei Medical University.

## COMPUTATIONS OF KEY ASPECTS OF DATA WITH ILLUSTRATIONS

### A.1. CALCULATION SURFACE AREA FOUR‐ANGLED AXIAL WALLS 2°, 6°, 12°, AND 18°

Stepwise trigonometric calculation for total surface area with H^3, 4, 5 mm^, taper angles M‐, D‐, B‐, Li‐walls at 2°, 6°, 12°, and 18° (see Figure 1; all four‐wall taper angles equal at each increment of taper categories)

M‐ and D‐wall areas with rectangle base width

$\frac{5\;\mathrm{mm}\times H^{5\;\mathrm{mm}}=\;\left[25\;{\mathrm{mm}}^{2}\right]\;}{\mathrm{COS}\;2{}^{\circ}}–\frac{\left[\mathrm{Two}\;\text{Right Triangles}\;\mathrm{ABC}\;½\;\left(H^{5\;\mathrm{mm}}÷\mathrm{TAN}\;2{}^{\circ}\right)\times 2\right]}{\mathrm{COS}\;2{}^{\circ}}$

B‐ and Li‐wall areas rectangle base width

$\frac{8\;\mathrm{mm}\times H^{5\;\mathrm{mm}}=\;\left[40\;{\mathrm{mm}}^{2}\right]\;}{\mathrm{COS}\;2{}^{\circ}}–\frac{\left[\mathrm{Two}\;\text{Right Triangles}\;\mathrm{ABC}\;½\;\left(H^{5\;\mathrm{mm}}÷\mathrm{TAN}\;2{}^{\circ}\right)\times 2\right]}{\mathrm{COS}\;2{}^{\circ}}$

### A.2. CALCULATION SURFACE AREA OCCLUSAL SURFACE

Occlusal surface area at taper angles M‐, D‐, B, Li‐walls at 2°, 6°, 12°, and 18° with H^3, 4, 5 mm^ (see Figure 1; all four‐wall taper angles equal at each increment of taper categories)

Rectangular cube occlusal area = base M–D width 5 mm × base B–Li width 8 mm = 40 mm^2^

Occlusal surface at 2° = area 40 mm^2^ – [4 × (H^5 mm^ Right Triangle ABC × TAN 2°)]

Area 40 mm^2^ – {[2 × (H^5 mm^ Right Triangle ABC × TAN 2°) + [2 × (H^5 mm^ Right Triangle ABC × TAN 12°)]}

### A.3. CALCULATION SMALL CONE HEIGHT AND LARGE CIRCLE BUR DIAMETER @ OCCLUSAL SURFACE LEVEL A.3. (SEE FIGURE 2)

Computation of tapered 172 bur and preparation heights and diameters:

1. Diameter of 172‐bur at tip base “known,” 1.18 mm
2. Calculate small cone length with known radius and external wall taper 3°

TAN3°=0.59÷xx=0.59/TAN3°=11.2mm

1. Total length of larger cone = 11.2 + 4 = 15.2 mm
2. Calculate diameter of large circle of 172‐bur at 4‐mm preparation height

TAN3°=x÷15.2x=15.2×TAN3°=1.6mm

### A.4. COMPUTATION LARGE AND SMALL 172‐BUR CONE AREAS, ONE‐HALF 172 BUR PREPARATION AREA A.4. (SEE FIGURE 3)

Calculate surface area of 172 bur external surface area = π × radius × slant length

First step, calculate slant length of large cone–slant length of small cone

Large cone slant length COS 3° = 15.2 ÷ x; large cone slant length = 15.2 ÷ COS 3° =

Small cone slant length OS 3° = 11.2 ÷ x; small cone slant length = 11.2 ÷ COS 3° =

Large cone 172 bur external surface area = π × 0.8 × 15.2 = 38.2

Small cone 172 bur external surface area = π× 0.59× 11.2 = 20.7

External 172 bur cone = 38.2 – 20.7 = 17.5 mm^2^

½ external surface area of 172 cone bur = 17.5 ÷ 2 = 8.8 mm^2^

### A.5. COMPUTATION TWO SIDES RECTANGULAR PORTION GROOVE PREPARATION AREA A.5. (SEE FIGURE 3)

Calculate surface area of rectangular portion of one vertical wall = ½ diameter of large circle × 4 mm vertical height at 3° [0.8× (COS 3°× 4)] = 3.2 mm^2^

Area of two vertical rectangular wall components = 2× 3.2 = 6.4 mm^2^

### A.6. COMPUTATION TWO SIDES RIGHT‐TRIANGLE GROOVE AREA A.6. (SEE FIGURE 3)

Calculate surface area of right triangles (two lateral rectangles and right triangles illustrated below):

Right triangle area = ½ × length of two sides adjacent the right angle

One side is height at 4 mm ÷ COS 3°

The other side is horizontal distance toward preparation finish line, using right triangle, ½ × (4 mm ÷ COS 3°) × (4 mm ÷ COS 3°) × [TAN (Preparation External Wall Taper Angle°)]

Two sides right triangle area at external wall taper angle 6° = 3.4 × 2 = 6.8 mm^2^

### A.7. COMPUTATION TWO‐GROOVE FLOOR AREAS A.7. (SEE FIGURE 4)

Calculation of the surface area of supplemental groove floor (illustrated in figures below):

Right triangle leg 0.4 + radius of large circle 0.8 = 1.2 × radius of large circle 0.59 = 0.71 mm^2^

½ Area of small 172 bur tip π × radius^2^ = ½ × π × (0.59)^2^ = 0.92 mm^2^

Total single groove area = ½ cone 8.8 + 2 rectangles 6.4 + 2 right triangles 6.8 + floor 0.71 = 22.7 mm^2^

### A.8. COMPUTATION TWO‐OCCLUSAL SURFACE AREAS LOST‐TO‐GROOVE PREPARATION A.8. (SEE FIGURE 5)

½ Area of large occlusal circle = ½ × π × (0.8)^2^ = 1.0 mm^2^

Remaining occlusal surface area lost = radius of large circle × diameter of large circle 0.8 × 1.6 = 1.3 mm^2^

Total occlusal surface area lost = 1.0 + 1.3 = 2.3 mm^2^

### A.9. COMPUTATION TWO‐AXIAL WALL SURFACE AREAS LOST‐TO‐GROOVE PREPARATION A.9. (SEE FIGURE 6)

Two right triangle areas = 2 × ½ [4 mm × (4 mm × TAN 3°)] = 1 × 4 mm × 0.2 mm = 0.8 mm^2^

Rectangle area = diameter of 172 bur tip (1.18 mm × 4 mm) = 4.72 mm^2^

Area 2 right triangles + 1 rectangle = 0.8 mm^2^ + 4.72 mm^2^ = 5.52 mm^2^ ÷ axial wall preparation angle adjustment COS 6° = total surface area lost lateral + occlusal = 2.3 mm^2^ + 5.52 mm^2^ = 7.82 mm^2^

Total area gained with two grooves = 2 × (22.7 mm^2^ − 7.82 mm^2^) = 30.52 mm^2^
